# Sex as an independent risk factor for cerebellar mutism syndrome: a validation study

**DOI:** 10.3389/fsurg.2025.1645832

**Published:** 2025-09-15

**Authors:** Xiaojiao Peng, Zhuo Zhi, Xinyi Chai, Hong Zhang, Yingjie Cai, Kaiyi Zhu, Nijia Zhang, Jia Wang, Hailang Sun, Guangheng Yin, Wenping Ma, Wei Yang, Ming Ge

**Affiliations:** ^1^ Department of Neurosurgery, Beijing Children’s Hospital, Capital Medical University, National Center for Children’s Health, Beijing, China; ^2^ Department of Image Center, Beijing Children’s Hospital, Capital Medical University, National Center for Children’s Health, Beijing, China; ^3^ Department of Cardiology, Shanxi Bethune Hospital, Shanxi Academy of Medical Sciences, Tongji Shanxi Hospital, Third Hospital of Shanxi Medical University, Taiyuan, China

**Keywords:** cerebellar mutism syndrome, sex differences, pediatric neurosurgery, posterior fossa tumor, risk factors

## Abstract

**Background:**

Cerebellar Mutism Syndrome (CMS) is a significant neurological complication following posterior fossa tumor surgery in children. The pathophysiological mechanisms of CMS remain elusive, and there is a growing interest in the potential influence of sex on its incidence. This study aims to evaluate sex as an independent risk factor for the development of CMS.

**Methods:**

A retrospective cohort study of 385 pediatric patients who underwent posterior fossa tumor surgery at Beijing Children's Hospital (2013–2024) was conducted. Comprehensive demographic, clinical, and pathological data were collected. Statistical analysis involved Chi-square tests for categorical variables, Kruskal–Wallis tests for non-parametric comparisons among groups, and logistic regression to identify independent predictors of CMS.

**Results:**

CMS occurred in 29.9% of all cases, with annual incidence ranging from 14.3% to 37.9%. Medulloblastoma was the most common pathology (38.4%), with a median maximal tumor diameter of 47.2 mm. Tumors were predominantly located at the midline (68.1%), and gross total resection was achieved in 86.3% of patients. Male patients exhibited a significantly higher incidence of CMS compared to females (73.0% vs. 53.0%, *p* = 0.003). Independent risk factors for CMS included male sex [OR 2.25; 95% CI (1.30–3.70)], midline tumor location [OR 7.47; 95% CI (2.79–19.98)], and medulloblastoma diagnosis [OR 2.11; 95% CI (1.24–3.59)].

**Conclusion:**

This study indicates a notable male predominance in CMS occurrence, suggesting the existence of sex-specific differences in cerebellar function and language development. These findings highlight the need for heightened monitoring and tailored interventions for male patients undergoing posterior fossa tumor surgery and suggest a potential biological basis for sex-specific differences in cerebellar function and vulnerability to surgical injury.

**Importance of the study:**

This study provides critical insights into the significant role of sex as an independent risk factor for Cerebellar Mutism Syndrome (CMS) following posterior fossa tumor surgeries in pediatric patients. By identifying male sex, midline tumor location, and medulloblastoma pathology as independent predictors, this research addresses a gap in understanding sex-based disparities in CMS development. These findings suggest potential gender-specific differences in cerebellar and language development, offering a foundation for future translational research and targeted clinical strategies. The results emphasize the need for heightened monitoring and tailored interventions, especially for male patients, to mitigate CMS risk and improve surgical outcomes in pediatric neurosurgery.

## Key points

1. Cerebellar mutism syndrome is significantly more common in male patients after posterior fossa tumor surgery.
2. Midline tumor location and medulloblastoma diagnosis are independent risk factors for cerebellar mutism syndrome.

## Introduction

Cerebellar Mutism Syndrome (CMS) is a common complication following following resection of posterior fossa tumors, affecting approximately 25% of this patient population (1, 2). Characterized by the sudden onset of mutism and emotional lability within 1–6 days post-surgery, CMS often persists for several months (3). Additional symptoms include ataxia, hypotonia, and irritability (4).

Although the exact pathogenesis of CMS remains elusive, the prevailing hypothesis attributes its development to injury of the deep cerebellar nuclei and their efferent pathways (5). Evidence implicates bilateral damage to the dentato-thalamo-cortical tracts in CMS pathogenesis (6). While midline tumor location and medulloblastoma have been recognized as risk factors (2, 7), the influence of age, sex, tumor size, hydrocephalus, and ventriculoperitoneal (VP) shunts remains controversial (8–10).

The role of sex in CMS development is not well understood. The literature presents mixed findings on the role of sex in CMS development. While some studies have suggested a possible male predisposition, other multicenter or review studies have reported no significant association or have shown conflicting results (3, 9, 11, 12). This discrepancy highlights a critical gap in our understanding and underscores the necessity for validation in large, well-characterized patient cohorts. Sex-based differences in the developmental trajectories of cerebellar function and language networks represent a compelling, yet unproven, hypothesis to explain possible sex disparities in CMS susceptibility across populations. Research indicates that girls exhibit superior expressive language skills compared to boys during the early development while boys tend to experience a relative delay in language acquisition and increased vulnerability to linguistic interference (13, 14). Functional MRI studies have also revealed sex-related differences in language processing, suggesting a potential link between sex and CMS susceptibility (15).

In this study, we aim to investigate the sex disparities in CMS incidence among pediatric patients undergoing posterior fossa tumor resection. Through multivariable and subgroup analyses, our goal is to discern definitive risk factors associated with CMS.

## Method and materials

### Study cohorts

This single-center, retrospective cohort study was conducted at Beijing Children's Hospital, National Center for Children's Health, Beijing, China. From July 2013 to March 2024, a total of 476 pediatric patients diagnosed with a posterior fossa tumor underwent surgical resection. Inclusion criteria were as follows: (1) age 0–18 years at the time of surgery; (2) a histologically confirmed posterior fossa tumor; and (3) surgical resection performed at our institution.

**Figure 1:**
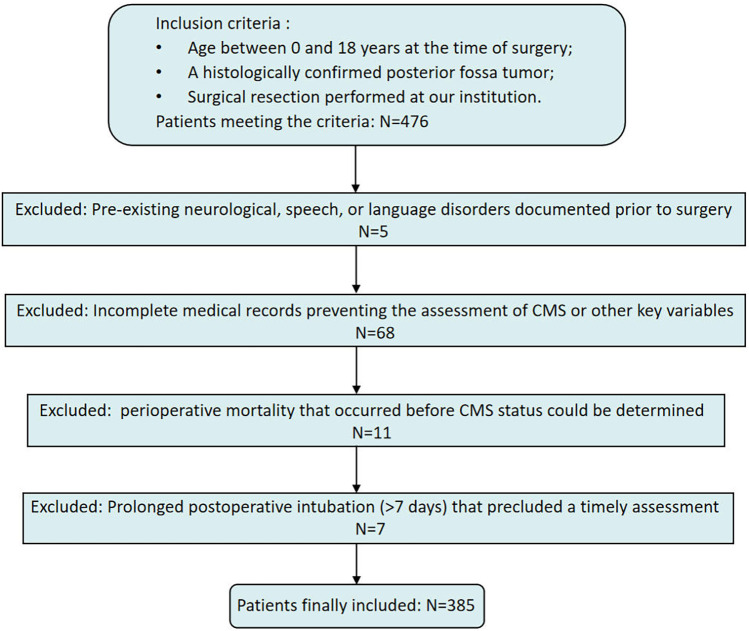
Patient inclusion and exclusion flowchart.

Exclusion criteria were as follows: (1) documented pre-existing neurological, speech, or language disorders documented; (2) incomplete medical records hat precluded assessment of CMS or other key variables; (3) perioperative mortality that occurred before CMS status could be determined; and (4) prolonged postoperative intubation (>7 days) that precluded a timely assessment for CMS. After applying these criteria, 385 patients were included in the final analysis (Figure 1).

All participants underwent a preoperative brain MRI. Data collection included demographic information, CMS status, radiological and histological characteristics, operative records, and follow-up data. CMS status was determined from clinical records or telephone interviews. Follow-up assessments were conducted every 6–12 months, either during outpatient visits or via telephone. During follow-up, data regarding the resolution of mutism were collected to determine the duration of symptoms for patients in the CMS group. Informed consent was obtained from all patients or their guardians, and the study was approved by the local Ethics Committee (Approval No. [2019-k-344).

### Diagnosis of CMS and follow-up

The diagnosis of CMS was determined according to the Iceland Delphi consensus diagnostic criteria, characterized by the delayed onset of impaired speech or complete mutism, accompanied by emotional lability, occurring after posterior fossa surgery (16).

The primary diagnosis was made by the neurosurgical team during the patient's hospitalization and documented in the clinical records. For cases where CMS status was ambiguous in the records, or for confirmation during follow-up, structured telephone interviews were conducted with the patients’ parents or legal guardians. The interview followed a standardized questionnaire designed to elicit parental recall of key symptoms in a non-leading and unbiased manner. The interview structure included the following components:

1. Timing and Onset: Questions to establish the timeline of any speech changes, specifically to identify a delay of 1–6 days post-surgery (e.g., “Thinking back to the week after the surgery, do you recall any changes in your child's ability to speak? If so, on which day did this change begin?”).
2. Nature of Speech Deficit: Questions to describe the severity of the speech change (e.g., “Could you describe the change? Did your child stop speaking completely, or were they just very quiet?”).
3. Emotional and Behavioral Changes: Probes for symptoms of emotional lability (e.g., “During that same period, did you notice any significant shifts in their mood, such as being unusually irritable, withdrawn, or crying more than usual?”).
4. Cross-Validation: A concluding question inquiring whether the term “Cerebellar Mutism Syndrome” or “Posterior Fossa Syndrome” was ever used by the clinical team, thereby validating the parent's descriptive recall against a formal diagnosis.

For pre-verbal patients (typically under 2 years of age), the diagnosis was based on a sudden and marked loss of previously acquired vocalizations (e.g., cessation of babbling or single-word use), combined with pronounced emotional lability (e.g., apathy, inconsolable crying) and other features of posterior fossa syndrome, as observed by both clinicians and parents.

### Definitions of interested variables

The analyzed variables included: age at surgery, sex, year of diagnosis, tumor size, tumor consistency (solid or non-solid), tumor location (midline or off-midline), presence of a VP shunt prior to resection, extent of resection [gross total resection (GTR) or Non-GTR], tumor pathology [medulloblastoma (MB) or Non-MB], Evans’ index, and presence of paraventricular and peritumoral edema.

Tumor size was determined by measuring the maximum diameter across axial, sagittal, and coronal MRI planes. Tumors were classified as non-solid if a cystic component accounted for more than 50% of the tumor volume. The categorization of tumor location was based on MRI findings, and the Evans’ index was calculated as the ratio of the maximum width of the frontal horns to the maximum internal width of the cranial cavity (normal range ≤0.30). Paraventricular and peritumoral edema were identified using T2-weighted or fluid-attenuated inversion recovery (FLAIR) MRI sequences. Surgical approach was categorized into four types: (1) Telovelar, (2) Transvermian, (3) Others, and (4) Unknown. MRI scans were reviewed and measurements were taken by two experienced neurosurgeons to ensure accuracy and consistency.

### Statistical methods

Statistical analyses were performed using python 3.7. Categorical variables were compared using the Chi-square test or Fisher's exact test, as appropriate. For continuous variables with non-normal distributions, the Kruskal–Wallis test was employed to compare median values across groups.

Univariable logistic regression was performed to identify potential risk factors for CMS. Variables with a *p*-value ≤ 0.10 in univariable analysis were entered into the multivariable logistic regression model. A stepwise selection procedure was used, with entry and removal criteria set at *p* = 0.01 and *p* = 0.1, respectively. The final model retained variables that were independently associated with CMS.

Collinearity among predictor variables was assessed using variance inflation factors (VIFs), with VIF > 5 indicating potential multicollinearity. Statistical significance was set at a threshold of *P* < 0.05 for all tests performed.

## Results

### CMS occurrence

**Figure 2:**
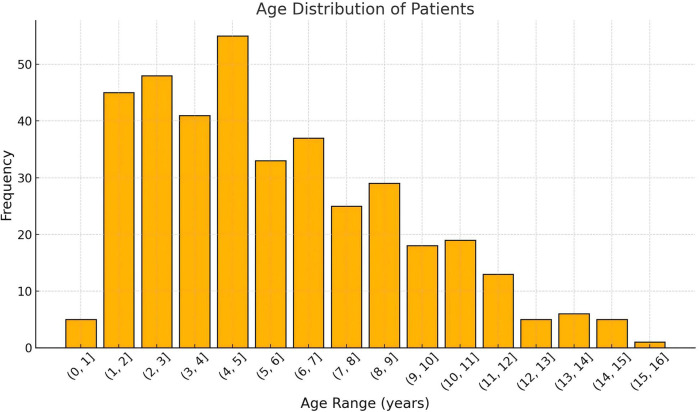
The overall age distribution of patients undergoing posterior fossa tumor surgery. The *x*-axis represents the age range in years, grouped in intervals of 1 year, while the *y*-axis indicates the number of patients in each age range. The histogram shows the frequency of patients in each age group, highlighting the predominant age ranges for surgical cases.

**Figure 3:**
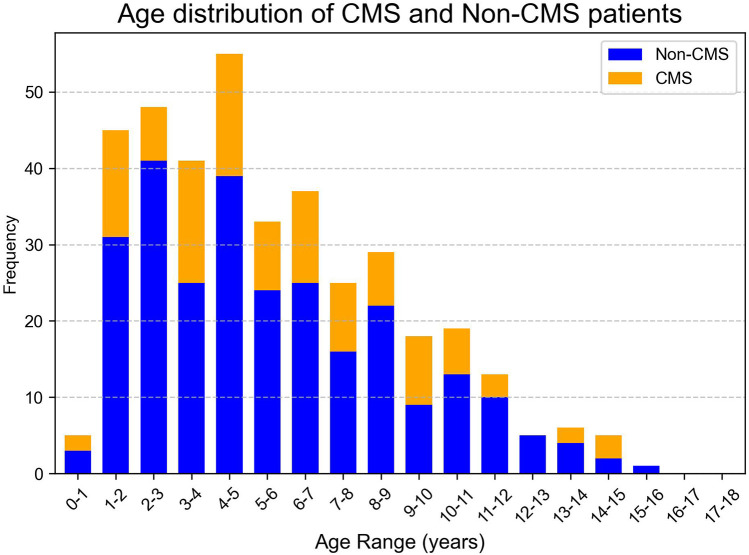
Age frequency distribution of CMS and Non-CMS groups. The *x*-axis represents the age range in years, grouped in intervals of 1 year, while the *y*-axis indicates the frequency as a proportion of the total number of patients in each group. The blue bars represent the CMS group, and the orange bars represent the Non-CMS group.

**Figure 4:**
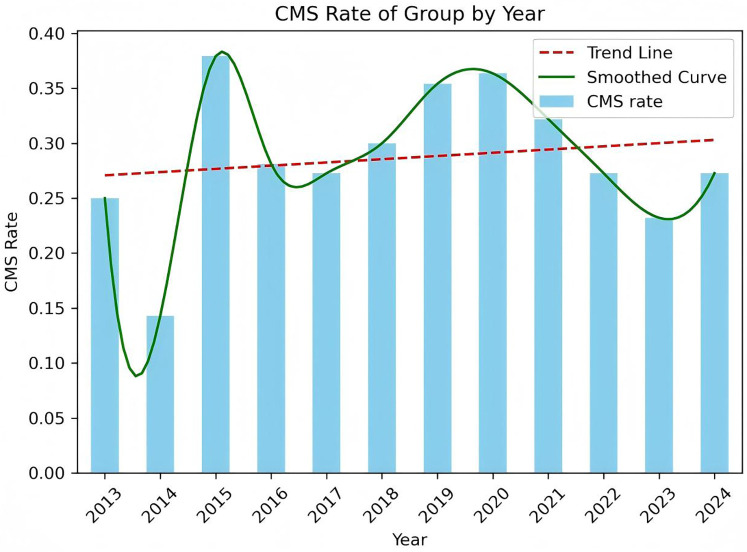
The trend of CMS occurrence from 2013 to 2024 in our medical center.

A total of 385 pediatric patients met the inclusion criteria and were analyzed. The cohort included 59.0% males, with a median age at surgery of 5.0 years (IQR: 3.0–7.9 years). The age distribution of the cohort, CMS and Non-CMS group were presented in Figures 2, 3. Tumors were predominantly located at the midline (68.1%), and gross total resection was achieved in 86.3% of cases. Medulloblastoma represented the most common pathology (38.4%), with tumors exhibiting a median maximal diameter of 47.2 mm (IQR: 39.1–55.2 mm). Preoperative VP shunt placement was performed in 9.1% of patients. CMS was observed in 29.9% of patients (115 out of 385). The incidence of CMS varied across study periods, ranging from 14.3% to 37.9% (Figure 4).

### Comparison of CMS and non-CMS group

The comparative analysis between the CMS (*n* = 115) and non-CMS (*n* = 270) groups revealed several statistically significant differences (Table 1). The CMS group exhibited a markedly higher proportion of male patients (73.0% vs. 53.0%; *p* < 0.001), midline tumor location (93.0% vs. 57.4%; *p* < 0.001), solid tumor texture (93.0% vs. 81.1%; *p* = 0.004), paraventricular edema (79.1% vs. 57.4%; *p* < 0.001), and medulloblastoma pathology (59.1% vs. 29.6%; *p* < 0.001). The adjusted Evans’ index was also significantly higher in the CMS group (median 3.2 vs. 2.8; *p* < 0.001). Additionally, the selection of surgical routes presented a significant difference between the CMS and Non-CMS groups, as detailed in Table 1.

**Table 1:** Group comparison of CMS and non-CMS.

Variables		Overall (*N* = 385)	Non CMS (*N* = 270)	CMS (*N* = 115)	*P*-value	Test
Sex, *n* (%)	Female	158 (41.0)	127 (47.0)	31 (27.0)	<0.001	Chi-squared
	Male	227 (59.0)	143 (53.0)	84 (73.0)		
Paraventricular edema, *n* (%)	No	139 (36.1)	115 (42.6)	24 (20.9)	<0.001	Chi-squared
	Yes	246 (63.9)	155 (57.4)	91 (79.1)		
Presurgical VP shunt, *n* (%)	No	350 (90.9)	250 (92.6)	100 (87.0)	0.117	Chi-squared
	Yes	35 (9.1)	20 (7.4)	15 (13.0)		
Midline location, *n* (%)	No	123 (31.9)	115 (42.6)	8 (7.0)	<0.001	Chi-squared
	Yes	262 (68.1)	155 (57.4)	107 (93.0)		
Tumor consistency, *n* (%)	No	59 (15.3)	51 (18.9)	8 (7.0)	0.005	Chi-squared
	Yes	326 (84.7)	219 (81.1)	107 (93.0)		
Extent of resection, *n* (%)	No	49 (12.7)	35 (13.0)	14 (12.2)	0.964	Chi-squared
	Yes	336 (87.3)	235 (87.0)	101 (87.8)		
Surgical Route, *n* (%)	1	86 (22.3)	53 (19.6)	33 (28.7)	<0.001	Chi-squared
	2	103 (26.8)	62 (23.0)	41 (35.7)		
	3	101 (26.2)	95 (35.2)	6 (5.2)		
	4	95 (24.7)	60 (22.2)	35 (30.4)		
MB, *n* (%)	No	237 (61.6)	190 (70.4)	47 (40.9)	<0.001	Chi-squared
	Yes	148 (38.4)	80 (29.6)	68 (59.1)		
Peritumoral edema, *n* (%)	No	267 (69.4)	191 (70.7)	76 (66.1)	0.432	Chi-squared
	Yes	118 (30.6)	79 (29.3)	39 (33.9)		
Age at surgery, median [Q1,Q3]		5.0 [3.0,7.9]	4.8 [2.7,7.8]	5.2 [3.3,8.2]	0.353	Kruskal–Wallis
Tumors size, median [Q1,Q3]		47.2 [39.1,55.2]	46.8 [37.7,55.4]	49.4 [42.3,55.1]	0.123	Kruskal–Wallis
Evans^a^10, median [Q1,Q3]		2.9 [2.5,3.5]	2.8 [2.4,3.3]	3.2 [2.7,3.8]	<0.001	Kruskal–Wallis

MB, medulloblastoma; EOR, extent of resection; 1, Telovelar approach; 2, Transvermian approach; 3, Others approach; and 4, Unknown approach.

^a^ The Evans’ Index is multiplied by 10 for analysis purposes.

No significant disparities were observed between the two groups regarding VP shunt placement, age at surgery, extent of resection, maximal tumor diameter, and the presence of peritumoral edema.

### Follow-up and symptom duration

Follow-up data on the duration of mutism were available for 98 of the 115 patients with CMS (85.2%). The median duration of mutism was 6.5 weeks [Interquartile Range (IQR): 3.0–12.0 weeks]. A comparison between sexes revealed no statistically significant difference in the duration of mutism (median 7.0 weeks for males vs. 6.0 weeks for females, *p* = 0.48, Kruskal–Wallis test).

### Univariable and multivariable analysis

Univariable analysis identified male sex [OR 2.37; 95% CI (1.50–3.76); *p* < 0.001], midline tumor location [OR 9.02; 95% CI (4.31–18.87); *p* < 0.001], MB pathology [OR 3.37; 95% CI (2.13–5.34); *p* < 0.001], paraventricular edema [OR 2.73; 95% CI (1.70–4.39); *p* < 0.001], solid tumor consistency [OR 3.23; 95% CI (1.43–7.29); *p* = 0.004], and the adjusted Evans’ Index [OR 1.07 per 0.01 increase; 95% CI (1.03–1.11); *p* = 0.001] as factors significantly associated with CMS (Table 2).

**Table 2:** Univariable logistic regression analysis for CMS.

Variables	Confident interval		Coefficient	Odds ratio	*p* value
	5%	95%			
Sex	0.402	1.354	0.878	2.406	0.000
Peritumoral edema	−0.251	0.682	0.216	1.241	0.365
Paraventricular edema	0.524	1.545	1.034	2.813	0.000
Presurgical VP shunt	−0.080	1.337	0.629	1.875	0.082
Midline location	1.537	3.053	2.295	9.923	0.000
Tumor consistency	0.356	1.916	1.136	3.115	0.004
EOR	−0.590	0.734	0.072	1.074	0.832
Surgical Route	−0.393	0.011	−0.191	0.826	0.064
MB	0.780	1.689	1.234	3.436	0.000
Evans^a^10	0.189	0.777	0.483	1.621	0.001
Age at surgery	−0.037	0.093	0.028	1.029	0.399
Tumors size	−0.004	0.030	0.013	1.013	0.125

^a^ The Evans’ index is multiplied by 10 for analysis purposes. MB, medulloblastoma; EOR, extent of resection.

**Table 3:** Multivariable logistic regression analysis for CMS.

Variables	Coefficient	Odds ratio	Confident interval		*p*-value	VIF
			5%	95%		
Constant	−3.27	0.04	−4.08	−2.47	0.000	
Midline location	2.01	7.47	1.23	2.79	0.000	2.38
Sex	0.79	2.20	0.28	1.30	0.003	1.83
MB	0.75	2.11	0.25	1.24	0.003	1.83

A stepwise logistic regression analysis was performed to identify independent risk factors for CMS. Variables with a *p*-value less than 0.01 were included in the model, and those with a *p*-value greater than 0.1 were excluded. The analysis revealed that male sex (OR = 2.20, 95% CI: 1.30–3.70, *p* = 0.003), midline tumor location (OR = 7.47, 95% CI: 2.79–19.98, *p* < 0.001), and MB pathology type (OR = 2.11, 95% CI: 1.24–3.59, *p* = 0.003) were independently associated with an increased risk of CMS. These results are detailed in Table 3. A collinearity analysis confirmed the absence of multicollinearity among these variables (VIF < 5 for all variables).

## Discussion

This study investigated the risk factors associated with CMS in pediatric patients undergoing posterior fossa tumor surgery, focusing on the role of sex, which has been a subject of debate with inconsistent findings across studies (17, 18). We found that male sex is an independent risk factor for CMS in a large Chinese cohort, along with midline tumor location and medulloblastoma pathology.

Our findings suggest that males are more susceptible to developing CMS. Importantly, this association persisted in our multivariable model after adjusting for both midline location and medulloblastoma pathology, indicating that the observed male predisposition is not solely explained by these established risk factors. Although the effect size for male sex is smaller than for midline location, this more than two-fold increased risk remains clinically relevant as a non-modifiable factor. Such findings can inform surgical planning, enhance parental counseling, and guide targeted postoperative monitoring strategies. The underlying mechanism may be related to sex-specific differences in language development and cerebellar function. The precise mechanism linking male gender to CMS is yet to be elucidated. We hypothesize that CMS is associated with language deficits stemming from brain development, with the cerebellum potentially playing a significant role in early language acquisition. The prevailing hypothesis attributes CMS to damage in the cerebral-cerebellar circuit; however, the specific functioning of this circuit in maintaining language and the cerebellum's role in speech processing remain obscure.

Several studies provide supporting evidence for our hypothesis: (1) A recent study indicates that children with CMS exhibit involvement in brain networks associated with Autism Spectrum Disorder (ASD) (19), highlighting similarities between the two language disorders. ASD is characterized by communication and social interaction deficits and shows a notable gender bias, with a male-to-female ratio of 4:1 (20). The enhanced plasticity theory posits that males have a lower threshold for developing an enhanced plastic response to environmental stimuli compared to females, which could affect specific brain regions involved in perception and language (21). (2) Girls tend to have an advantage over boys in the early stages of language acquisition (22). Girls outperform boys in word production tasks during early language development, with this disparity widening with age. However, by school age, the gender effect on language performance diminishes and may vanish (23). Yet, previous studies have not consistently identified males as an independent risk factor for CMS (2, 18, 24). The discrepancy may be attributed to differences between Chinese and Western language systems, as phonological processing in English and Chinese engages distinct brain regions (25). Chinese phonological processing recruits the middle frontal cortex, motor cortex, and supplementary motor area in the left hemisphere (26), whereas for native English speakers, it engages the left inferior prefrontal cortex and superior temporal gyrus (27). Furthermore, research has shown that language processing differences between children and adults differs developmentally between language, indicating developmental effects on the language network (28). This evidence may explain why male gender was not identified as an independent risk factor in some studies.

In our investigation, we have identified midline tumor location and MB as independent risk factors for CMS. Extensive literature supports the idea that midline location is associated with an increased risk of CMS, whereas tumors located in the cerebellar hemispheres are linked to a protective effect and seldom progress to CMS. While midline location was a powerful predictor in our model, we did not specifically analyze brainstem invasion as a variable. We acknowledge this as a limitation. However, it is a clinical reality that the large midline tumors typically seen in this pediatric population (median diameter 47.2 mm in our cohort) almost invariably lead to brainstem compression. Thus, the “midline location” variable likely reflects the significant risk associated with brainstem involvement. MB is recognized as a significant and established risk factor for CMS, with recent findings highlighting associations a correlation between the molecular subtypes of MB and the development of CMS. Specifically, the Wingless, Group 3, and Group 4 subtypes are associated with a notably higher incidence of CMS than the sonic hedgehog subtype independent of other risk factors. Additionally, a compelling body of evidence has emerged linking CMS with the malignancy grade of tumors, with MB exhibiting a higher grade in comparison to astrocytoma and ependymoma. Despite the majority of MBs being midline in location, the interplay between MB and midline positioning has not been extensively explored. Utilizing stepwise logistic regression analysis in our study, we have determined that both midline location and MB independently contribute to the risk of CMS. Furthermore, our collinearity analysis has confirmed the absence of multicollinearity among the variables examined.

Beyond the clinical risk factors identified in this large cohort, the specific neural structures damaged during surgery are paramount in the development of CMS. The reviewer rightly points to the importance of features like superior cerebellar peduncle (SCP) and dentate nucleus (DN) involvement. Indeed, our group has previously published a detailed analysis of postoperative MRI features in a cohort from our institution (29). That study demonstrated that postoperative injury to the bilateral SCPs and DNs, quantified as a cerebro-cerebellar circuit injury score on T2-weighted MRI, was a powerful independent predictor of both the occurrence and duration of CMS. The current study complements these detailed radiological findings by validating, in a larger and more heterogeneous population, that male sex stands as an independent clinical risk factor alongside broad predictors like midline location and medulloblastoma pathology.

In our study, we investigated the association of various factors, including age at the time of surgery, maximum tumor diameter, presence of paraventricular edema, and Evan's index, with CMS. However, none of these variables showed an independent association with CMS. The link between age and CMS remains enigmatic. Our results align with several previous studies (2, 9, 30), which also reporting no significant association between age and CMS. In contrast, other studies have identified a younger age as an independent risk factor for CMS (17, 31–33). This discrepancy in findings might be due to sample variability or potential biases in the diagnosis of CMS. Further research is warranted to elucidate the relationship between age at the time of surgery and the development of CMS.

The association between Evans’ index and CMS remains controversial. Although Tian et al. proposed that a rapid decrease in intracranial pressure may contribute to CMS development (34), a meta-analysis did not uncover any significant association between CMS and hydrocephalus (18). This finding is further supported by Keating et al., who observed no significant difference in cerebrospinal fluid (CSF) diversion between the CMS and non-CMS groups (35), which corroborates our results. Despite these findings, the role of certain risk factors remains ambiguous and necessitates future validation. Establishing a rigorous, explicit, practical, and universally applicable definition of CMS, potentially through multicenter collaboration, could address these ambiguities. As Wickenhauser proposed, defining specific diagnostic criteria for CMS would enhance both research and clinical practice (36).

Our data presented a significant year-to-year variation in CMS incidence, which ranged from 14.3% to 37.9%. During 2013–2014, the patient cohort size was particularly small (*n* < 10), which lead to the statistical instability and larger fluctuations in the CMS rates. In the subsequent period, when the annual patient numbers were larger and more stable, the CMS incidence rate also stabilized, generally fluctuating between 25% and 35%.

Our study, however, has limitations. Firstly, it is a single-center, retrospective study, which may limit the generalizability of our findings. Secondly, data on factors such as handedness, medulloblastoma molecular subtypes, and surgical approaches were incomplete and not included in the analysis. Third, while our group has previously published on the importance of detailed postoperative radiological features like superior cerebellar peduncle and dentate nucleus injury in predicting CMS (29), a detailed re-analysis of these specific features for the entire 385-patient cohort was beyond the scope of the current clinical validation study. Furthermore, data on brainstem invasion, medulloblastoma molecular subtypes, intraoperative technologies such as intraoperative imaging and neuromonitoring were not consistently available across the study period and were therefore not included in our analysis. These factors represent important areas for future prospective, multicenter studies. Finally, the lack of a universally adopted, stringent definition for CMS remains a challenge for the entire field.

## Conclusion

This study demonstrates that male sex is an independent risk factor for developing CMS in pediatric patients undergoing posterior fossa tumor surgery. Along with midline tumor location and medulloblastoma pathology, male sex contributes to increased CMS susceptibility. The identification of male sex as an independent risk factor, despite a moderate effect size, underscores its value for risk stratification and future mechanistic studies. The intrinsic mechanisms driving this sex-based disparity in CMS susceptibility are yet to be fully elucidated. One plausible explanation may lie in the divergent developmental milestones of language acquisition between the sexes, potentially predisposing males to a greater risk of CMS. Further research is needed to elucidate the mechanisms behind this sex disparity and to develop targeted interventions.

## Data Availability

The raw data supporting the conclusions of this article will be made available by the authors, without undue reservation.
